# Synthesis of Butyl-β-D-Galactoside in the Ternary System: Acetone/1-Butanol/Aqueous Solution

**DOI:** 10.3389/fbioe.2020.00859

**Published:** 2020-07-23

**Authors:** Diego Ahumada, Felipe Arenas, Fabián Martínez-Gómez, Cecilia Guerrero, Andrés Illanes, Carlos Vera

**Affiliations:** ^1^Laboratory of Molecular Microbiology, Department of Biology, Faculty of Chemistry and Biology, Universidad de Santiago de Chile, Santiago, Chile; ^2^School of Biochemical Engineering, Faculty of Engineering, Pontificia Universidad Católica de Valparaíso, Valparaíso, Chile

**Keywords:** lactose, alkyl-glycoside, β-galactosidase, butyl-β-galactoside, sugar-based surfactants

## Abstract

The enzymatic synthesis of short-tailed alkyl glucosides is generally carried out in an aqueous-organic biphasic reaction medium with a rather low fatty alcohol concentration in the aqueous phase (where the synthesis occurs). Thus, hydrolytic reactions have a significant impact on the synthesis performance. Given this background, the use of acetone as cosolvent was studied for the synthesis of butyl-β-galactoside with *Aspergillus oryzae* β-galactosidase. The liquid–liquid equilibrium of the reaction mixture components (acetone/1-butanol/aqueous solution) was determined and the single- and two-phase regions were defined at 30, 40, and 50°C. It was observed that the liquid–liquid equilibrium of the ternary system acetone/1-butanol/water differs significantly from the one obtained using an aqueous solution (50 mM McIlvaine buffer pH 4.5; 5 g L^–1^) instead of water. This is mainly because of the salting-out effect of the buffer; nevertheless, the presence of lactose also altered the equilibrium. Having this in mind, the effects of temperature (30 and 50°C) and reaction mixture composition were assessed. Three general conditions were evaluated: single-phase ternary system (30% acetone), two-phase ternary system (10% acetone) and two-phase binary system (0% acetone). Acetone had a deleterious effect on enzyme stability at 50°C, leading to low reaction yields. However, no enzyme deactivation was detected at 30°C. Moreover, a reaction yield of 0.98 mol mol^–1^ was attained in the 30/50/20% (w/w) mixture of acetone/1-butanol/aqueous solution. This very high yield can be explained by the huge increase in the concentration of 1-butanol and the reduction of water activity. The synthesis was carried out using also the β-galactosidase immobilized in glyoxal-agarose and amino-glyoxal-agarose, and by aggregation and crosslinking. In the case of agarose-derived catalysts, two average particle diameters were assessed to evaluate the presence of internal mass transfer limitations. Best yield (0.88 mol mol^–1^) was obtained with glyoxal-agarose derivatives and the particle size had non-effect on yield. The chemical structure of butyl-β-galactoside was determined by NMR and FT-IR.

## Introduction

Alkyl-glycosides (AGs) are a new family of non-ionic surfactants that stand out from conventional non-ionic surface agents for being easily biodegradable, non-toxic and hypoallergenic (Rather and Mishra, 2013). Furthermore, they are synthesized from renewable raw materials, so they are a sound replacement for nonyl-phenol and its ethoxylated-derivatives (United States Environmental Protection Agency, 2012). AGs have been commonly used as ecofriendly substitutes of conventional surfactants in cleaning, lubrication, wetting, emulsification and foaming (Mańko and Zdziennicka, 2015); nevertheless, they have also gained attention due to their intrinsic properties. For instance, AGs have been posed as replacement of the polysorbates used to prevent the aggregation of therapeutic proteins. AGs are much more chemically stable than polysorbates, so they do not provoke unwanted modifications in therapeutic proteins during their storage (Maggio, 2012; Mahjoubi et al., 2017).

AGs are formed by a carbohydrate (polar head) coupled by an ether linkage (*O*-glycosidic bond) to an alkyl chain (non-polar tail). At industrial scale AGs are produced by Fischer glycosidation, i.e., the condensation of a fatty alcohol and a carbohydrate using an acid catalyst and high temperature to favor substrate miscibility (Balzer and Lueders, 2000). AGs can be synthetized also with glycoside hydrolases as biocatalysts, with the advantages of using mild reaction conditions, reducing waste production and not requiring highly pure substrates (Van Rantwijk et al., 1999; Vera et al., 2020). However, enzymatic synthesis has some drawbacks, such as a lower yield than obtained in chemical synthesis. Yield typically decreases as molecular weight of the fatty alcohol increases (Rather and Mishra, 2013) so that the enzymatic production of AGs is only efficient for those having a short tail (Hronská et al., 2016; Vera et al., 2017a,b,c). Since AGs having tails with more than 8 carbon atoms are required for most industrial applications (Gaudin et al., 2019), the synthesis of AG with shorter tails has been relatively less reported. However, in recent years the production of short-tailed AGs has received more attention due to their interesting tensioactive and antimicrobial properties (Charoensapyanan et al., 2016; Garcia-Arellano et al., 2019). Furthermore, they can be used as building-blocks for organic synthesis (Wang et al., 2019). Recently, Starchem Enterprises (China) has started the bulk production of butyl and hexyl-glucosides, marketing both as solubilizers in hard surface cleaning, especially in beer bottle cleaning and metal anti-corrosion cleaning (Starchem Enterprises Limited, 2019). Therefore, this renewed interest in short-tailed AGs production opens up an opportunity for using biocatalysis and underutilized carbohydrates in the synthesis of ecofriendly surfactants.

The enzymatic synthesis of AGs is usually based on the capability of glycoside hydrolases to transfer a carbohydrate moiety to a nucleophile containing a hydroxyl group, in this case, to a fatty alcohol (Rather and Mishra, 2013; Vera et al., 2020). Thus, to favor the synthesis of AGs (transglycosylation) over the hydrolysis of the donor substrate, a fatty alcohol concentration as high as possible is required. However, fatty alcohols are slightly miscible or immiscible with aqueous solutions, so the enzymatic synthesis of AGs generally occurs in a biphasic system (Vera et al., 2017b), which aqueous phase has a poor concentration of the fatty alcohol. On the other hand, mass transfer phenomena and partitioning may play an important role in the reaction performance. AGs are expected to be preferentially partitioned into the organic phase, avoiding its hydrolysis (secondary hydrolysis) and in this way favoring AGs accumulation (Vera et al., 2017a, b).

In this work, the enzymatic synthesis of butyl-β-galactoside from 1-butanol and lactose was studied considering that lactose is an underutilized, inexpensive and readily available carbohydrate (Chen and Gänzle, 2017). Furthermore, this synthesis can be efficiently catalyzed by β-galactosidases, which are robust commodity enzymes that are commercially available for the food industry at a rather low price (Illanes, 2011; Vera et al., 2017b). Butyl-glycosides have interesting applications in cleaning formulations (Starchem Enterprises Limited, 2019) and as building-blocks for more complex surfactants (Monsan et al., 1996). In the present work, the use of acetone is assessed in order to increase the concentration of 1-butanol in the aqueous phase and avoid the formation of a biphasic reaction medium. In this manner, it is expected to favor the transgalactosylation reaction, so increasing the reaction yield. However, this strategy may have some problems that needs to be experimentally addressed, e.g., the absence of the organic phase may promote the (secondary) hydrolysis of the butyl-β-galactoside produced and the acetone may significantly reduce the enzyme stability and the solubility of lactose. Given the complexity of the experimental system, the surface response methodology is typically used to determine the effect of the experimental variables and optimize their value to maximize an objective function (Vera et al., 2017b, c). However, this approach provides a limited understanding of the phenomena underlying behind. So, in this article, a deterministic experimental approach was utilized.

## Materials and Methods

*Aspergillus oryzae* β-galactosidase (Enzeco^®^Fungal Lactase) was donated by Enzyme Development Corporation (EDC, New York, NY, United States). The enzyme preparation was stored refrigerated, with no appreciable change in its activity during the research period. 6% BCL Agarose Bead Standard and 6% BCL Agarose Bead Fine were provided by Agarose Bead Technologies (ABT, Madrid, Spain). All the remaining reagents were of analytical grade (or superior) and were purchased to Merck (Darmstadt, Germany) or Sigma (St. Louis, MO, United States). Before being used acetone and 1-butanol were dehydrated using 3Å molecular sieves.

### Liquid–Liquid Equilibrium for the Mixture Acetone/1-Butanol/Aqueous Solutions

The liquid–liquid equilibrium for the mixture acetone/1-butanol/aqueous solutions was studied at 30, 40, and 50°C for the following aqueous solutions: water, 50 mM McIlvaine buffer pH 4.5 and a 5 g L^–1^ dissolution of lactose in 50 mM McIlvaine buffer pH 4.5. The experimental procedure followed was the one reported by Palei (2010). Briefly, acetone was gently dropped into mixtures of 1-butanol/aqueous solutions until reaching the miscibility point, where the mass percentage of each component was calculated and reported using a triangular (ternary) plot. The experiments were done at least in triplicate and for all the reported data the coefficient of variation (standard deviation to mean ratio) was lower than 5%. It is worth mentioning that high lactose concentrations were not evaluated because the formation of a precipitate was observed at certain experimental conditions. The lower temperature limit was selected considering 5°C above the room temperature for allowing an adequate control of the temperature by the thermocirculator (Julabo Corio CD, Germany). To minimize the effect of enzyme inactivation during testing, the upper limit was selected by taking 5°C below the optimum temperature for the enzyme reported by the supplier.

### Synthesis of Butyl-β-Galactoside With Soluble Enzyme in a Ternary System

Synthesis of butyl-β-galactoside was performed at 30 and 50°C with soluble *A. oryzae* β-galactosidase, using lactose as donor substrate, 1-butanol as acceptor substrate and acetone as cosolvent. An initial total mass of 40 g was used for all the assays; all assays were conducted in triplicate. Mean values and standard deviation of the triplicates are reported in all cases. Reactions were carried in 100 mL Schott bottles contacting different ratios of an aqueous solution (5 g L^–1^ lactose in 50 mM McIlvaine buffer pH 4.5), 1-butanol and acetone. The combined effect of three mass percentages of aqueous solution (20, 40, and 60%) and three mass percentages of acetone (0, 10, and 30%) were experimentally evaluated, the remaining mass percentages corresponding to 1-butanol. These conditions were chosen considering the results shown in section “Characterization of the Ternary System Acetone/1-Butanol/Aqueous Solutions.” Three general conditions were evaluated: single-phase ternary system (30% acetone), two-phase ternary system (10% acetone) and two-phase binary system (0% acetone). In this way, the effect of cosolvent concentration and the number of liquid phases could be studied. Syntheses were initiated adding a constant enzyme load of 400 IU. Samples of 0.5 mL were taken at regular time intervals during 2 h. Reaction was stopped by vigorous mixing of the samples with an equal volume of 75 mM Na_2_CO_3_. Then, the samples were vacuum dried in a centrifugal concentrator Speedvac SPD111 VP2 (Thermo Scientific) in order to remove the organic solvents, since they interfere with butyl-β-galactoside quantification. Afterward, the samples were reconstituted in MiliQ water and filtered using a disposable PDVF syringe filter (diameter: 13 mm; pore: 0.22 μm) provided by Filterpore (Chile). Carbohydrates (lactose, glucose and galactose) and butyl-β-galactoside were determined by HPLC.

The synthesis performance was evaluated using the yield (*Y*) and productivity (π) of butyl-β-galactoside as parameters:

(1) $$Y=\frac{B\,G}{L\,a\,c}$$

(2) $$\pi =\frac{B\,G}{V\cdot t}$$

where *Y* represents the moles of butyl-β-galactoside obtained per mol of lactose added into the reaction and π corresponds to the volumetric productivity of synthesis, namely the moles of butyl-β-galactoside obtained per unit of reaction volume and unit of time. Since the synthesis of butyl-β-galactoside is a kinetically controlled reaction both parameters were evaluated when the maximum concentration of the product was reached.

### Immobilization of *A. oryzae* β-Galactosidase

In a previous report about the synthesis of butyl-β-galactoside with immobilized enzymes, some indirect evidences of internal diffusional restrictions (IDR) were observed (Vera et al., 2017b). In the present work, agarose beads were used as support with different average particle diameters for determining the influence of IDR in *Y* and π. Also, two different functionalized agaroses were used: glyoxal-agarose (GA) and amino-glyoxal agarose (Am-GA). 6% BCL Agarose Bead Standard and 6% BCL Agarose Bead Fine with particle diameters ranging from 50 to 150 and from 20 to 50 μm, respectively, were employed as starting raw material. Aside, the use of β-galactosidase crosslinked aggregates (CLAGs) prepared using 1-propanol as precipitating agent was assessed. Because of the very high specific activity of CLAGs the presence of IDR is to be expected.

Immobilization in GA was conducted as previously reported (Guerrero et al., 2017a, b). Firstly, the agarose beads were activated with glycidol and then oxidized with sodium (meta)periodate. Secondly, the enzyme was linked to the activated support using an enzyme load of 30 mg_protein_ g^–1^_support_, 0.1 M bicarbonate buffer pH 10 with 20% (v/v) of glycerol. The suspension was kept at 4°C under gentle stirring. Afterward, the Schiff base formed between glyoxal and amine groups was reduced to a secondary amine with sodium borohydride. Then, the biocatalyst was recovered by filtration and washed with 50 mM McIlvaine buffer pH 4.5. On the other hand, the immobilization in Am-GA was conducted following the method described by Guerrero et al. (2017a). Briefly, the support was activated with triethylamine and then oxidized with sodium (meta)periodate. An enzyme dissolution (30 mg_protein_ g^–1^_support_) in 5 mM phosphate buffer pH 7 was contacted with the activated support for 2 h at 25°C. The suspension was filtered and the solid resuspended in 5 mM bicarbonate buffer pH 10 (with 20% v/v glycerol). The suspension was kept overnight under gentle stirring at 4°C. The Schiff base formed between glyoxal and amine groups in the enzyme was reduced using sodium borohydride. Finally, the biocatalyst was recovered by filtration and washed with 50 mM McIlvaine buffer pH 4.5. The β-galactosidase crosslinked aggregates (CLAGs) were prepared as described by Guerrero et al. (2015, 2020). The enzyme was precipitated in 1-propanol 50% (v/v) and the precipitated protein was crosslinked using glutaraldehyde as bifunctional reagent. Glutaraldehyde was added at a ratio of 5.5 g per gram of protein and the suspension was kept under gentle stirring during 5 h. Then, the biocatalyst was recovered by centrifugation and washed with McIlvaine buffer 50 mM pH 4.5.

To assess the net outcome of the immobilization process, the immobilization yield (percentage of contacted activity expressed in the enzyme-derivative) and the specific activity of the biocatalysts (IU g_biocatalyst_^–1^) were determined. The immobilization yield of β-galactosidase on GA standard, GA fine, Am-GA standard, Am-GA fine and CLAGs were 27, 50, 29, 44, and 81%, respectively. The specific activity of GA standard, GA fine, Am-GA standard, Am-GA fine and CLAGs were 2,364 ± 45, 4,614 ± 98, 2,618 ± 38, 3,253 ± 61 and 40,267 ± 785 IU g^–1^, respectively.

### Synthesis of Butyl-β-Galactoside With Immobilized Enzyme

Reactions were carried out using a magnetically stirred glass reactor with a total volume of 250 mL provided by Pobel (Spain). Synthesis were conducted at the best conditions reported in section “Synthesis of Butyl-β-Galactoside in a Ternary System,” i.e., 30°C, 30/50/20% (w/w) of acetone/1-butanol/aqueous solution (5 g L^–1^ lactose in 50 mM McIlvaine buffer pH 4.5), respectively. An initial total mass of 100 g was used for all the assays and reactions were started by adding 1,000 IU of immobilized β-galactosidase, in order to maintain the same enzyme load (10 IU per gram of reaction medium) than in the synthesis conducted with the free enzyme. Samples of 1 mL were taken at regular time intervals during 2 h. In order to remove the catalyst, samples were centrifuged for 30 s using a spin centrifuge (BiosebLab, France) and then the supernatant was filtered using a disposable PDVF syringe filter (diameter: 13 mm; pore: 0.22 μm) provided by Filterpore (Chile). Quantification of substrate and products was conducted as described in section “Synthesis of Butyl-β-Galactoside With Soluble Enzyme in a Ternary System.” All assays were conducted in triplicate. Mean values and standard deviation of the triplicates are reported in all cases.

### Determination of β-Galactosidase Activity

One international unit (IU) of β-galactosidase was defined as the amount of biocatalyst that hydrolyzes 1 μmol of *o*-nitrophenol-β-D-galactopyranoside (ONPG) per minute at 40°C and pH 4.5. The *o*-nitrophenol produced was measured using a Jasco V-730 spectrophotometer provided with temperature control and a magnetic stirring system. 50 mM McIlvaine buffer was used to set the pH at 4.5. A specific activity of 108.5 ± 1.6 IU mg^–1^ was determined for the commercial preparation of *Aspergillus oryzae* β-galactosidase.

### Substrates and Products Determination by High Performance Liquid Chromatography (HPLC)

Lactose, glucose, galactose, butyl-β-galactoside, acetone, and butanol were determined using an HPLC system (Jasco, Japan), consisting on a refractive index detector RI-4030, a quaternary pump 4180, a column heater CO-4060, an autosampler AS 4050, and interphase LCNETII-ADC. The peaks were integrated using the Chromnav 2.0 software provided by the manufacturer. Samples were eluted through an Aminex^®^ HPX-87H (300 mm × 7.8 mm) column at a flow rate of 0.4 mL min^–1^. Mobile phase was a mixture of 0.005N sulfuric acid and 0.2% (v/v) of acetonitrile. Column and detector were kept at constant temperatures of 45 and 40°C, respectively. Retention times for lactose, glucose, galactose and butyl-β-galactoside were 10.9, 12.8, 13.7, and 19.5 min, respectively. Acetone, and butanol were quantified employing the same procedure, but using an eluent flow rate of 0.5 mL min^–1^. Their retention times were 26.1 and 42.9 min, respectively.

### Purification and Identification of Butyl-β-Galactoside

Butyl-β-galactoside was synthesized by scaling the procedure described in section “Synthesis of Butyl-β-Galactoside With Soluble Enzyme in a Ternary System” to 1 kg of reaction mixture. However, in this case the reaction was stopped by boiling. The reacted mixture was concentrated to 100 mL (approximately) in a rotary evaporator at 65°C. The concentrate was fully dried at room temperature in a Speedvac SPD 111 VP2. Butyl-β-galactoside was purified by liquid extraction. Powder was dissolved in 10 mL of 0.1M NaOH and contacted with 300 mL of 1-butanol. The aqueous phase was discarded and 10 mL of 0.1M NaOH was added to the organic phase. This procedure was repeated six times and then repeated three times but replacing the NaOH solution by MilliQ water. Finally, the organic phase was dried at room temperature in a Speedvac SPD 111 VP2. Butyl-β-galactoside was obtained with a purity over 99.9% (Checked by HPLC) and used as HPLC-standard.

Butyl-β-galactoside was characterized by ^1^H and ^13^C nuclear magnetic resonance (NMR) using a Bruker NMR Spectrometer, Neo Multinuclear Advance 400 MHz. Samples were dissolved in D_2_O and experiment conducted at 300 K. Also, butyl-β-galactoside was analyzed using a Fourier Transform Infrared (FT-IR) spectrometer FT-IR IFS 66V BRUKER. A KBr pellet was prepared and its spectrum was recorded in the range from 400 to 4,000 cm^–1^. All the analyses were performed by the Analytical Service of the Faculty of Chemistry and Biology of the University of Santiago of Chile.

### Statistical Analyses

All statistical analyses were done using Microsoft Excel 365 tools and a significance level of 0.05 was utilized for the analysis of variance (Anova), *t*-paired and Tukey tests. All experiments were done at least in triplicate. Mean and standard deviation are reported in all cases. Superscripts in Tables 1 and 2 are used to indicate a statistically non-significant difference according to Tukey test.

**TABLE 1:** Product yield (*Y*) and productivity (π) of butyl-β-galactoside synthesis with soluble β-galactosidase from *A. oryzae* in acetone/1-butanol/aqueous solution ternary system.

**Temperature (°C)**	**Mass percentage**			**Reaction parameters**	
	**Acetone**	**1-butanol**	**Aqueous solution**	***Y* (mol mol^–1^)**	**π (mM h^–1^)**
30	0	80	20	0.69 ± 0.06^a^	2.47 ± 0.09^a^
	10	70	20	0.80 ± 0.001^a^	3.54 ± 0.13^b^
	30	50	20	0.98 ± 0.04	1.81 ± 0.05^a,c^
	0	60	40	0.41 ± 0.03^b^	3.37 ± 0.20^b^
	10	50	40	0.57 ± 0.03^a,c^	1.52 ± 0.21^c,d^
	30	30	40	0.58 ± 0.03^a,c^	1.49 ± 0.22^c–e^
	0	40	60	0.39 ± 0.002^b,d^	3.37 ± 0.11^b^
	10	30	60	0.28 ± 0.01^b,d,e^	1.35 ± 0.24^c–f^
	30	10	60	0.23 ± 0.01^e^	1.18 ± 0.18^c–f^
50	0	80	20	0.55 ± 0.01^m^	5.82 ± 0.33
	10	70	20	0.44 ± 0.01^m,n^	0.99 ± 0.02^m^
	30	50	20	0.58 ± 0.06^m^	0.87 ± 0.12^m,n^
	0	60	40	0.39 ± 0.001^n,o^	3.12 ± 0.24
	10	50	40	0.13 ± 0.02^p^	0.87 ± 0.12^m,n^
	30	30	40	0.12 ± 0.01^p,q^	0.48 ± 0.09^m,n,p^
	0	40	60	0.29 ± 0.01^o^	1.37 ± 0.02^m,n^
	10	30	60	0.03 ± 0.001^p,q,r^	0.26 ± 0.05^p,q^
	30	10	60	0.09 ± 0.004^p,q,r^	0.69 ± 0.06^m,n,p,q^

*Superscripts are used to indicate a statistically non-significant difference according to Tukey test (α = 0.05).*

**TABLE 2:** Product yield (*Y*) and productivity (π) of butyl-β-galactoside synthesis with immobilized β-galactosidase from *Aspergillus oryzae* in acetone/1- butanol/aqueous solution ternary system.

**Support**	**Reaction parameters**	
	***Y* (mol mol^–1^)**	**π (mM h^–1^)**
GA standard	0.87 ± 0.03^a^	1.14 ± 0.006
GA fine	0.87 ± 0.04^a,b^	0.83 ± 0.02^a^
Am-GA standard	0.78 ± 0.02^a,b^	0.74 ± 0.04^a^
Am-GA fine	0.81 ± 0.04^a,b^	0.78 ± 0.004^a^
CLAGs	0.74 ± 0.03^b^	1.64 ± 0.02

*Syntheses were conducted at 30°C and 30/50/20% (w/w) in acetone/1-butanol/aqueous solution ternary system. Superscripts are used to indicate a statistically non-significant difference according to Tukey test (α = 0.05).*

## Results and Discussion

### Characterization of the Ternary System Acetone/1-Butanol/Aqueous Solutions

**FIGURE 1:**
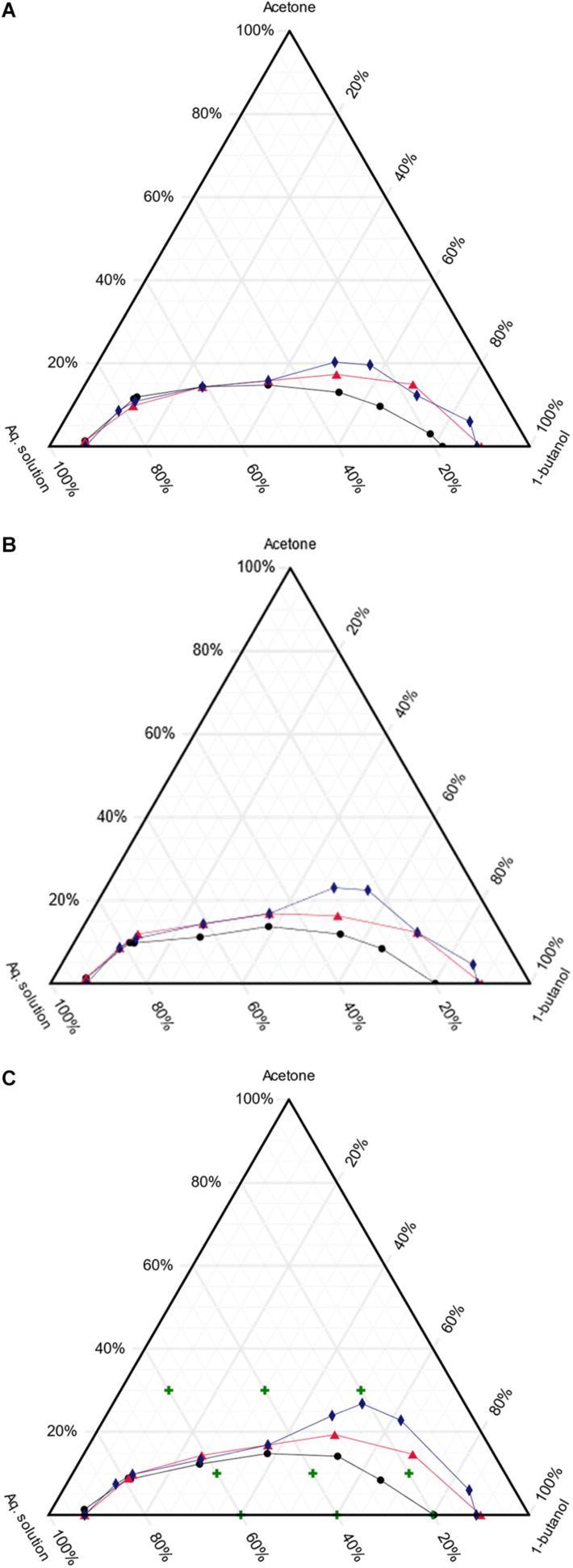
Ternary phase diagram for the acetone/1-butanol/aqueous solution system. **(A)**, **(B)**, and **(C)** correspond to 30, 40, and 50°C, respectively. Black circles: water; red triangles: 50 mM McIlvaine buffer pH 4.5 and blue diamonds: lactose solution 5 g/L in 50 mM McIlvaine buffer pH4.5. The green crosses in panel **(C)** correspond to the experimental conditions studied in this work for the synthesis of butyl-β-galactoside. The mixture composition is expressed in mass percentages.

Even though the liquid–liquid equilibrium for the system acetone/1-butanol/water has been previously reported, the presence of ionic solutes in the aqueous phase significantly modifies the liquid–liquid equilibrium (Santos et al., 2001; Palei, 2010). The addition of electrolytes may provoke the “salting-out” phenomena, namely the reduction of the mutual solubility of the solvents, so increasing the two-phase region. Also, it may produce a decrease in the solubility of a non-electrolyte in the aqueous-phase (Santos et al., 2001; Palei, 2010). In the particular case of butyl-β-galactoside synthesis, the presence of the buffer components (electrolytes) and the carbohydrates (non-electrolytes) alter the liquid–liquid equilibrium for the system acetone/1-butanol/water. Since it is difficult to forecast the resulting equilibrium, this was experimentally assessed. As can be appreciated in Figure 1, the electrolytes forming the McIlvaine buffer exert a salting-out effect, which is reflected by an increase in the two-phase region, this effect being more pronounced at higher temperatures and in the presence of lactose. Also, Figure 1 indicates that the salting-out effect is present in the binary system 1-butanol/water, leading in this case to a lower content of water in the organic phase, while the composition of the aqueous phase does not vary significantly. The obtained results are in good agreement with those reported by Palei (2010), who studied the effect of salts (KCl and NaCl) in the ternary system acetone/1-butanol/water.

Considering that the effect of salting-out was more severe at 50°C, the experimental conditions to evaluate the synthesis of butyl-β-galactoside in the ternary system acetone/1-butanol/water were defined taking into account Figure 1C. In this figure the selected conditions are represented by green crosses. Three general conditions were assessed: single-phase ternary system (30% acetone), two-phase ternary system (10% acetone) and two-phase binary system (0% acetone). In this way, the effect of cosolvent concentration and the number of liquid phases was determined.

### Synthesis of Butyl-β-Galactoside in a Ternary System

**FIGURE 2:**
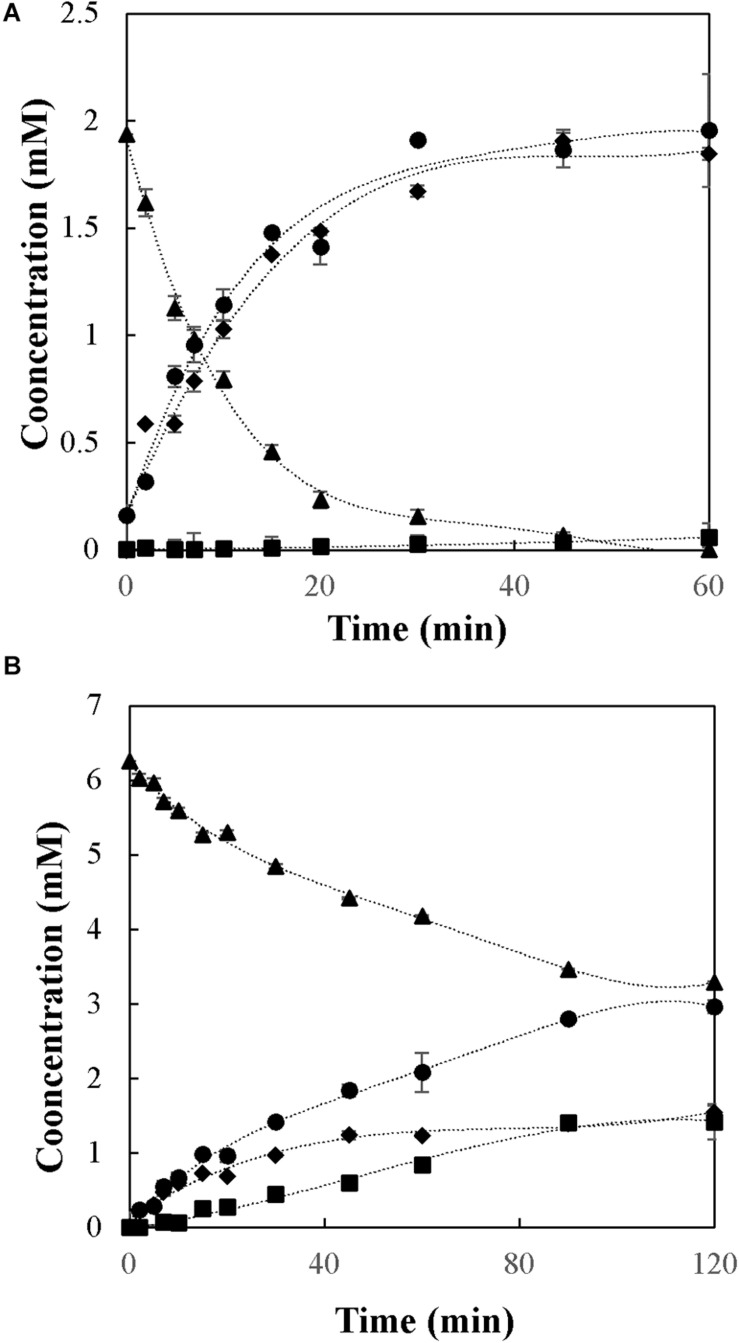
Synthesis of butyl-β-galactoside with soluble β-galactosidase from *A. oryzae* in the ternary system acetone/1-butanol/aqueous solution. **(A)** 30/50/20 and **(B)** 30/10/60% (w/w) of acetone/1-butanol/aqueous solution (5 g L^–1^ of lactose). Reactions were conducted at 30°C. Triangles: lactose; circles: glucose; diamonds: butyl-β-galactoside; squares: galactose.

As an example of the reaction behavior, the profiles of the donor substrate and the products in the synthesis of butyl-β-galactoside are presented in Figure 2, where subfigures A and B show the kinetics of the synthesis under the best and worst experimental conditions in terms of *Y*. In this figure, the disappearance of lactose is accompanied by an equimolar appearance of glucose, because the latter corresponds to the leaving group of the donor substrate (lactose). Depending on the reaction conditions, the galactose moiety was preferably transferred either to 1-butanol or water to produce butyl-β-galactoside (Figure 2A) or galactose (Figure 2B), respectively.

The results obtained in the synthesis of butyl-β-galactoside in the ternary system: acetone/1-butanol/aqueous solution are presented in Table 1. Under the studied conditions, lower values of *Y* and π were obtained at 50°C than at 30°C. In most of the experiments conducted at 50°C, negligible changes in the concentration of substrates and products were observed after 30 min of reaction, which is due to enzyme inactivation. In a previous work Vera et al. (2017b) reported a negative effect of temperature on yield; however, the temperature effect was less severe in that case. Brena et al. (2003) reported that acetone, dioxane, and ethanol have a deleterious effect on the stability of this enzyme even at low concentrations. Therefore, the results in Table 1 can be explained considering that in the present work acetone, which was used as a cosolvent, probably reduced the thermal stability of *A. oryzae* β-galactosidase. Despite this disadvantage of using acetone as a co-solvent, it is worth mentioning that acetone also has desirable properties: it is miscible with water, it is an aprotic solvent that cannot act as an acceptor in transgalactosylation reactions, whereby unwanted parallel reactions are avoided, it can be produced from renewable raw materials, and it is generally classified as a green solvent (Joshi and Adhikari, 2019). In addition, the adverse effect of acetone on enzyme stability decreases significantly at 30°C. As can be seen in Table 1, a *Y* value close to 1 mol mol^–1^ was attained at 30°C in the presence of 30% (w/w) acetone. This *Y* value is remarkably higher than the values reported by Vera et al. (2017b) and Ismail et al. (1999), where maximum values of 0.58 and 0.79 mol mol^–1^ were obtained, respectively. Also, it is higher than reported for the closely related synthesis of propyl-β-galactoside (Hronská et al., 2016; Vera et al., 2017c).

If the synthesis of butyl-β-galactoside is conducted at a temperature low enough to avoid enzyme inactivation, the effect of acetone can be understood considering two limiting cases of very low and very high ratios of aqueous phase to organic phase (acetone plus 1-butanol). The former case is illustrated by the results obtained at 30°C and 20% (w/w) of aqueous solution (see Table 1); under this condition, the addition of the cosolvent provoked an increase in the 1-butanol concentration in the phase where reaction occurred. The initial concentration of 1-butanol was 76.4 ± 1.5 and 596 ± 5.2 g L^–1^ at 0/80/20 and 30/50/20 mass percentages of acetone/1-butanol/aqueous solution, respectively. This impressive increase is due to the system passing from two-phase to single-phase. Also, there is an obvious decrease in water activity. The increase in the acceptor substrate concentration and the reduction in water activity altogether led to the almost fully suppression of the hydrolytic reaction. In the latter case, which is depicted by the experiments at 60% (w/w) of aqueous solution, the rise in the acceptor substrate concentration and the reduction in water activity were not enough to compensate for the lower enzyme stability. Likewise, Lang et al. (2006) reported a compromise between the gain in *Y* and the loss in enzyme stability, when an ionic liquid was used as a cosolvent in transgalactosylation reactions. In that report the authors concluded that the moderate increase in *Y* (10%) justifies the use of an ionic liquid in terms of practical considerations (productivity and cost). Similarly, in the synthesis of alkyl-glycoside with the α-amylase from *Thermotoga maritima* the use of deep eutectic solvents (DES) provoked a reduction in enzyme stability and a decrease in enzyme activity, but favored transglycosylation (alcoholysis) over hydrolysis at concentration lower than 10% v/v (Miranda-Molina et al., 2019). In the synthesis of hexyl-β-galactoside with *A. oryzae* β-galactosidase (Vera et al., 2017a), the addition of 30% (v/v) acetone produced a significant increase in *Y*, mainly because of a decrease in secondary hydrolysis. On the other hand, when butyl-β-galactoside synthesis was conducted in a biphasic media (1-butanol/aqueous solution), the butyl-β-galactoside was partitioned preferentially into the organic phase (Vera et al., 2017b). So, a reduction in the secondary hydrolysis (product hydrolysis) was expected. Considering the results obtained at 30/50/20% (w/w) of acetone/1-butanol/aqueous solution, it is concluded that an increase in 1-butanol concentration in the phase where reaction occurs and the reduction in water activity have a stronger effect than the partition effects on increasing *Y*. For *A. oryzae* β-galactosidase, this may be due to the fact that the Michaelis constant for butyl-β-galactoside is 57-fold lower than the one for lactose (Vera et al., 2017a), so butyl-β-galactoside is preferentially hydrolyzed at the aqueous phase.

### Synthesis of Butyl-β-Galactoside Using Immobilized β-Galactosidase

In this report, three methodologies of immobilization were done to produce the catalysts that were assessed for the synthesis of butyl-β-galactoside in the ternary system acetone/1-butanol/aqueous solution. Immobilization in GA beads was evaluated because the best yield in the synthesis of butyl-β-galactoside in a binary system was obtained using this catalyst (Vera et al., 2017b). Guerrero et al. (2017a) immobilized *A. oryzae* β-galactosidase in GA, Am-GA, carboxyl-glyoxal agarose and copper (II) chelate-glyoxal-agarose beads attaining the higher specific activity and stabilization factor with the enzyme immobilized in Am-GA, so based in this background information, the immobilization of the enzyme in Am-GA was also evaluated. Vera et al. (2017b) also studied the synthesis of butyl-β-galactoside with GLAGs produced using ammonium sulfate as precipitating agent obtaining poor results. Recently, Guerrero et al. (2020) greatly improved the manufacture of CLAGs by precipitating *A. oryzae* β-galactosidase with organic solvents, obtaining highly active and stable CLAGS when using 1-propanol as precipitating agent. Therefore, this type of catalyst was evaluated in the synthesis of butyl-β-galactoside.

**FIGURE 3:**
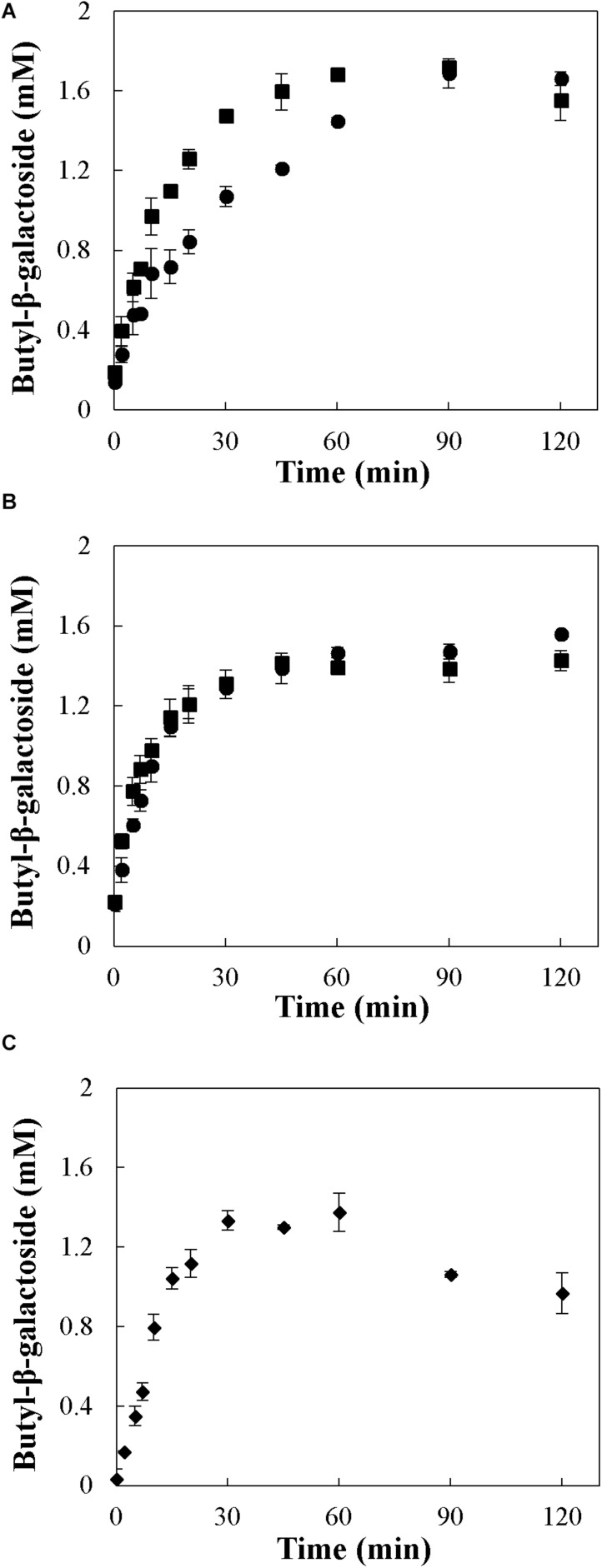
Synthesis of butyl-β-galactoside with immobilized β-galactosidase from *A. oryzae* in the ternary system acetone/1-butanol/aqueous solution. Reactions were conducted at 30°C and 30/50/20% (w/w) of acetone/1-butanol/aqueous solution (5 g L^–1^ of lactose). Enzyme was immobilized in panels **(A)** GA, **(B)** Am-GA, and by CLAGs **(C)**. Circles: standard agarose; squares: fine agarose.

Table 2 presents the *Y* and π values for the synthesis of butyl-β-galactoside with *A. oryzae* β-galactosidase immobilized in four different supports and CLAGs. The Anova test for the results in Table 2 indicates that the catalyst has a statistically significant effect on *Y* (*p*-value <0.02) and π (*p*-value <0.002). Tukey test for *Y* (α = 0.05) indicates that only a “honestly significant difference” exists between the result obtained with GLAGs and with the enzyme immobilized in GA standard and fine. Also, Tukey test (α = 0.05) indicates that only π values for GLAGs and GA standard are different between them and with respect to other values of π in. Table 2. Considering only the mean of each parameter, best *Y* values were obtained using mono-functional GA as support (Figure 3A), followed by hetero-functional Am-GA (Figure 3B) and CLAGs (Figure 3C). Similar results were reported by Vera et al. (2017b), who obtained the best *Y* values using mono-functional GA as support. These authors reported a *Y* of 0.76 mol mol^–1^ using *A. oryzae* β-galactosidase immobilized in GA, 25°C, 70% (v/v) 1-butanol and 30% (v/v) of aqueous solution. In the present work, a 14.5% higher *Y* value was obtained as a consequence of the use of acetone as cosolvent, which allowed the reaction to be carried out in a single-phase medium and favored the reaction of synthesis by increasing the concentration of 1-butanol and decreasing the water activity.

he literature for the syntheVera et al. (2017b) suggested that the synthesis of butyl-β-galactoside with *A. oryzae* β-galactosidase immobilized in GA standard may be subjected to IDR. For this reason, in the present work, the enzyme was also immobilized in GA fine, which has a much lower average diameter than the standard one. If the reaction of synthesis is subjected to IDR, a lower effect is expected as the catalyst diameter decreases (so that the Thièle modulus decreases). Figure 3A shows that the rate of butyl-β-galactoside production was higher when GA standard is used instead of GA fine. Since t-paired test indicates that both product patterns are statistically different (*p*-value <0.01), it is concluded that the catalyst diameter modifies the reaction kinetics. One plausible explanation for this behavior it is the presence of IDR. In a kinetically controlled reaction, IDR reduces to a major extent the effectiveness factor of the reaction with higher Thièle modulus, favoring the competing reactions. So, in this case, IDR favor the synthesis of the glycoside by depressing the rate of hydrolysis more than the rate of synthesis. The same behavior has been observed in the synthesis of the peptide kyotorphin with α-chymotrypsin immobilized in GA, where IDR reduced the effectiveness factor of the reaction of hydrolysis to a larger extent than the reaction of synthesis (Bahamondes et al., 2017). On the other hand, no significant differences (*p*-value <0.001) were observed between the synthesis with *A. oryzae* β-galactosidase immobilized in Am-GA standard and Am-GA fine (see Figure 3B). This suggests that the different enzyme orientation in Am-GA catalyst relieves the impact of IDR. Since both GA and Am-GA are based on the same material, small changes in the diffusing component should be expected and the observed differences between both catalysts might be the result of different intrinsic kinetic parameters. In this regard, Guerrero et al. (2018) demonstrated that GA standard, GA fine and Am-GA standard have an apparent Michaelis constant for lactose of sevenfold, threefold and fivefold the value of the Michaelis constant of the free enzyme, respectively. Furthermore, Hoffmann et al. (2020) demonstrated that the milieu inside the catalyst particle exerts a significant influence on the selectivity of the reaction, i.e., the ratio between the rate of synthesis and the rate of hydrolysis. In the synthesis of propyl-glucoside with immobilized β-glucosidase from *Thermotoga neapolitana*, the functionalization of off-stoichiometric thiol-ene (OSTE) materials with malonic acid and imidazole groups increased the selectivity twofold to threefold. Besides that, lower *Y* values were obtained with the agarose-derived catalysts (Table 2) with respect the ones obtained with the free enzyme (Table 1). This may be due to the hydrophilic milieu inside the agarose derived supports, which enhances the hydrolysis rate (Mateo et al., 2007).

The values shown in Table 2 are among the highest reported in the literature for the synthesis of alkyl-glycosides with immobilized enzymes. For instance, Hronská et al. (2016) attained *Y* values around 0.46 and 0.40 mol mol^–1^ for the synthesis of ethyl- and propyl-β-galactoside with *A. oryzae* β-galactosidase entrapped in Lentikat^®^. Kumar et al. (2017) only reported the capability of the β-glucosidase from *Streptomyces griseus* immobilized onto zinc oxide nanoparticles to synthetize propyl-, butyl-, pentyl-, hexyl- octyl-, benzyl- and 2-phenyl-ethyl glucosides. Gargouri et al. (2004) evaluated Duolite, Amberlite, Cellite and DEAE-Sepharose as supports for the immobilization of *Sclerotinia sclerotiorue* β-xylosidase. The free enzyme catalyzed the synthesis of alkyl-xylosides from xylan and fatty alcohols of 4–8 carbons. However, the Cellite derivative was the only one able to catalyze the synthesis of an alkyl-xylosides (hexyl-xyloside). Woudenberg-van Oosterom et al. (1998) studied the synthesis of galactopyranosyl-glycerol with *A. oryzae* β-galactosidase immobilized in Duolite supports, reporting that the higher *Y* values were obtained by the transgalactosylation route, reaching a *Y* value of 0.7 mol mol^–1^ with lactose as donor substrate.

### Butyl-β-Galactoside Characterization

**FIGURE 4:**
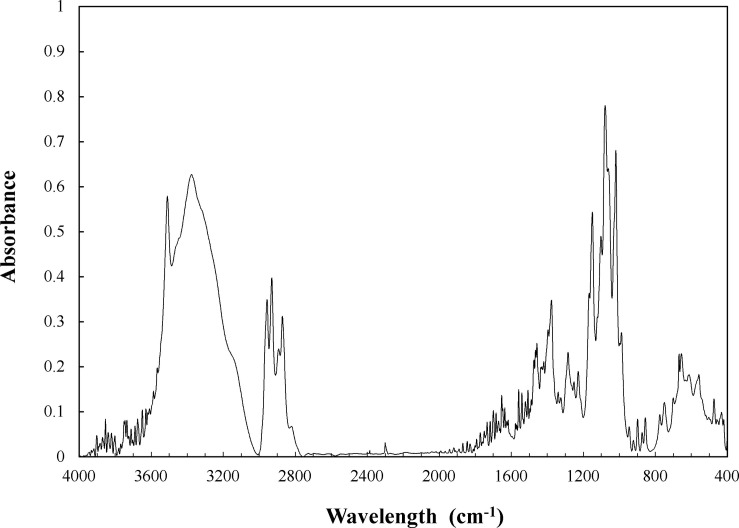
FT-IR spectrum for butyl-β-galactoside. Conditions: KBr pellet, 298 K and atmospheric pressure.

Butyl-β-galactoside FT-IR spectroscopy is shown in Figure 4. The characteristic absorption of O-glycosidic bond (ether linkage C-O-C) was observed as a small peak at 1,735 cm^–1^. The symmetric and asymmetric bending of C–H bonds are shown as peaks at 1,377 and 1,466 cm^–1^, respectively. The symmetric/asymmetric stretching of C–H bonds is visualized in the region from 2,800 to 3,000 cm^–1^. Peaks at 2,872/2,929 cm^–1^ and 2,890/2,956 cm^–1^ correspond to CH_2_ and CH_3_ groups. The O–H bond (mainly from the galactose moiety) is observed as a wide band in the 3,000–3,600 cm^–1^ range. Thus, these signals confirm the presence of a carbohydrate residue linked by *O*-glycosidic bond to an alkyl chain in the purified compound. Furthermore, the FT-IR spectrum obtained for butyl-β-galactoside is in full agreement with those reported for dodecyl/tetradecyl-glucoside (Gustianthy et al., 2019) and isooctyl-glucoside (Zou et al., 2016).

**FIGURE 5:**
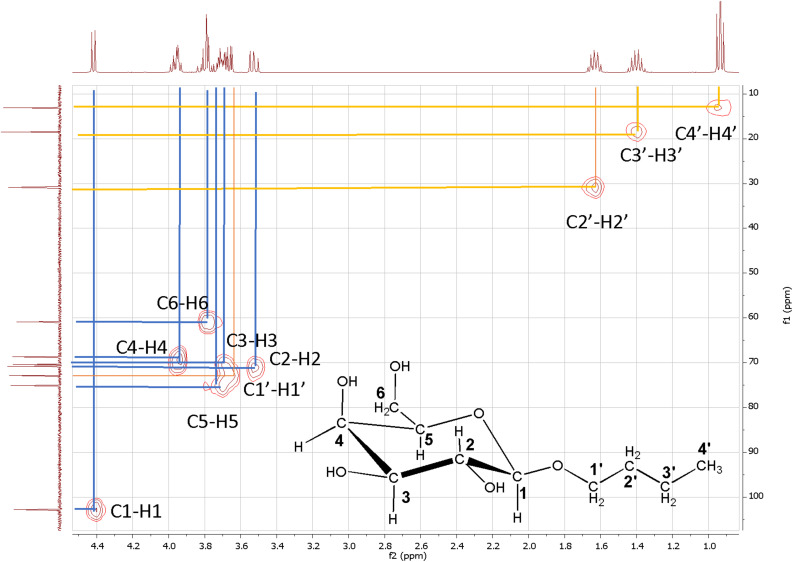
Bidimensional NMR spectrum ^13^C-^1^H HSQC for butyl-β-galactoside.

NMR spectrum of butyl-β-galactoside (see Table 3) shows two groups of signals characteristic for carbohydrates, between 5.2 and 3.6 ppm in the ^1^H NMR and between 103 and 65 ppm in the ^13^C NMR spectrum. A signal at 4.45 ppm is shown in the ^1^H NMR spectrum, which is characteristic of the anomeric protons in axial configuration (β anomer). This is further supported for the coupling constant whose value is 7.9 Hz. No signals were detected around 5 ppm ruling out the existence of the α anomer. This result confirms that no mutarotation exists, which indicates that the butyl group is directly bound to the oxygen in the anomeric carbon forming an acetal group (Bubb, 2003; Irazoqui et al., 2009). Below 2 ppm, three signals are shown characteristic of a hydrocarbon chain: a quintuplet at 1.63 ppm ascribed to a methylene group, a sextuplet at 1.4 ppm ascribed to a methylene and a triplet at 0.93 ppm ascribed to a methyl group. Ten major signals are observed in the ^13^C NMR spectrum, from which six are characteristic of a pyranosyl group and four correspond to a hydrocarbon chain. At 102.73 ppm a signal is observed ascribed to the anomeric carbon, in which the hydrogen atom is bound in axial position. The other five signals of the pyranosic ring are at 75.09, 70.79, 70.20, 68.66, and 60.88 ppm. There are four signals tan can be ascribed to a butyl group at 72.76, 30.9, 18.47, and 13.08 ppm, from which the first three can be ascribed to methylene groups and the fourth to a methyl group. The signal at 72.76 ppm correspond to the carbon directly linked to the oxygen atom in the pyranosic ring (Bubb, 2003; Charoensapyanan et al., 2016; Zou et al., 2016). For the complete ascription of the observed signals, ^1^H-^1^H COSY and ^13^C-^1^H HSQC bidimensional spectroscopy was used. Results for the ascribed signals are shown in Figure 5 and Table 3.

**TABLE 3:** ^1^H and ^13^C Nuclear magnetic resonance for butyl-β-galactoside.

**^1^H NMR**									
**Pyranosic ring δ (ppm) (J/Hz)**						**Alkyl group δ (ppm) (J/Hz)**			
	
**H-1**	**H-2**	**H-3**	**H-4**	**H-5**	**H-6**	**H-1′**	**H-2′**	**H-3′**	**H-4′**
4.45	3.54	3.68	3.96	3.72	3.8	3.68	1.63	1.4	0.93
(d, 1H	(dd, 1H	(dd, 1H	(dd, 1H	(m, 1H	(d, 1H	3,98	(q, 2H	(sx, 2H	(t, 2H
J = 7.9)	J = 7.7)	J = 3.4)	J = 3.9)	J = 4.2)	J = 4.6)	(2, 2H	J = 7.3)	J = 7.3)	J = 7.3)

**^13^ C NMR**									

**Pyranosic ring δ (ppm)**						**Alkyl group δ (ppm)**			
	
**C-1**	**C-2**	**C-3**	**C-4**	**C-5**	**C-6**	**C-1′**	**C-2′**	**C-3′**	**C-4′**

102.73	70.79	70.2	68.68	75.09	60.88	72.76	30.9	18.47	13.08

*d, doublet; t, triplet; q, quintuplet; sx, sextuplet; dd, doublet of doublets; m, multiplet.*

Results obtained in this section are in full agreement with the catalytic mechanism of β-galactosidases (Vera et al., 2020). This enzyme is classified as a retaining enzyme, namely, one that retains the anomeric configuration of the donor substrate in the product, so that a β-glycosidic bond between the galactose moiety and the butyl group was expected. Also, the condensation of the hydroxyl group in 1-butanol with the anomeric carbon in the galactose moiety was expected as result of the transgalactosylation reaction.

## Conclusion

The effect of the main operating variables (temperature and reaction mixture composition) on the enzymatic synthesis of butyl-β-galactoside in the ternary system acetone/1-butanol/aqueous solution was elucidated. Furthermore, the best combination of these variables was determined in order to maximize *Y*. To do so, the first step was to study the liquid–liquid equilibrium of the reaction mixture components, defining the single and two-phase regions. Afterward, the synthesis of butyl-β-galactoside was conducted at three general conditions: single-phase ternary system, two-phase ternary system and two-phase binary system (0% acetone). In this way, the effect of cosolvent concentration and the number of liquid phases was determined. Acetone proved to be highly inactivating for the enzyme at 50°C. However, this effect was not perceived at 30°C. At the latter temperature and 30/50/20% (w/w) of acetone/1-butanol/aqueous solution a *Y* value close to 1 mol mol^–1^ was obtained for the free enzyme. This was due to the transition from a two-phase to a single-phase system, which provoked a huge increase in the 1-butanol concentration and a reduction in the water activity, leading to an almost complete suppression of the hydrolytic reactions. The use of a cosolvent for increasing the miscibility of the fatty alcohol is not new and usually has shown to have a negative impact on *Y* and π. Thus, the merit of this research lies in the successful use of acetone as cosolvent, which is a cheap and environmentally friendly organic solvent. When the immobilized enzyme was used lower *Y* values were obtained. Since mass transfer limitations were discarded, it is hypothesized that the hydrophilic milieu of agarose reduced the reaction selectivity. This issue will be addressed in further studies by evaluating different supports and functionalizations. The chemical structure of butyl-β-galactoside was determined by NMR and FT-IR, being in full agreement with the expected one from mechanistic considerations.

## Data Availability Statement

The datasets presented in this article are not readily available because they will be available only for non-profit related purposes. Requests to access the datasets should be directed to CV, carlos.vera.v@usach.cl.

## Author Contributions

DA carried out most experiments. FM-G conducted the NMR analyses and the remaining authors contributed to the experimental design and writing of the manuscript. All authors contributed to the article and approved the submitted version.

## Conflict of Interest

The authors declare that the research was conducted in the absence of any commercial or financial relationships that could be construed as a potential conflict of interest.

## Abbreviations

π: volumetric productivity (mM h^–1^)
AG: alkyl-glycoside
Am-GA: amino-glyoxal agarose
BG: maximum concentration of butyl- β-galactoside during the synthesis (mol L^–1^)
CLAGs: β-galactosidase crosslinked aggregates
FT-IR: Fourier-transform infrared spectroscopy
GA: glyoxal-agarose
IDR: internal diffusional restrictions
Lac: initial lactose concentration (mol L^–1^)
NMR: nuclear magnetic resonance
*t*: reaction time (h)
*V*: volume time (L)
*Y*: reaction yield (mol mol^–1^).
